# A Path Planning Method with Perception Optimization Based on Sky Scanning for UAVs

**DOI:** 10.3390/s22030891

**Published:** 2022-01-24

**Authors:** Songhe Yuan, Kaoru Ota, Mianxiong Dong, Jianghai Zhao

**Affiliations:** 1 Institute of Advanced Manufacturing Technology, HeFei Institutes of Physical Science, Chinese Academy of Sciences, Changzhou 213164, China; 2 Department of Information and Electronic Engineering, Muroran Institute of Technology, Muroran 050-8585, Hokkaido, Japan; ota@mmm.muroran-it.ac.jp (K.O.); mxdong@mmm.muroran-it.ac.jp (M.D.); 3 HeFei Institutes of Physical Science, Chinese Academy of Sciences, Hefei 230031, China; jhzhao@iamt.ac.cn

**Keywords:** unmanned aerial vehicle (UAV), path planning, scene understanding

## Abstract

Unmanned aerial vehicles (UAVs) are frequently adopted in disaster management. The vision they provide is extremely valuable for rescuers. However, they face severe problems in their stability in actual disaster scenarios, as the images captured by the on-board sensors cannot consistently give enough information for deep learning models to make accurate decisions. In many cases, UAVs have to capture multiple images from different views to output final recognition results. In this paper, we desire to formulate the fly path task for UAVs, considering the actual perception needs. A convolutional neural networks (CNNs) model is proposed to detect and localize the objects, such as the buildings, as well as an optimization method to find the optimal flying path to accurately recognize as many objects as possible with a minimum time cost. The simulation results demonstrate that the proposed method is effective and efficient, and can address the actual scene understanding and path planning problems for UAVs in the real world well.

## 1. Introduction

**Figure 1:**
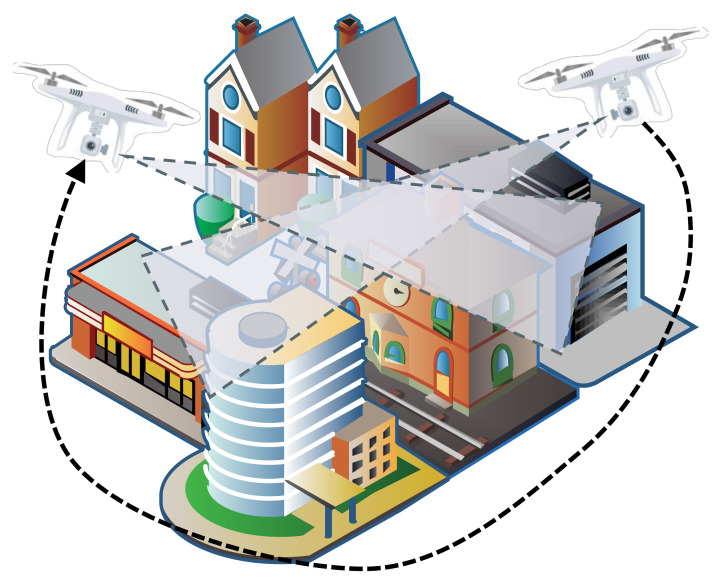
Path Planning for Scene Understanding with UAVs.

The unmanned aerial vehicle (UAV) is often used in disaster management. For example, after a disaster, people can use the UAV to detect the disaster area to find the damaged buildings and residents; and in the disaster recovery stage, the UAV can be used to assess the severity of the disaster, the feasibility, and cost of the reconstruction. They are utilized to assist people in disaster reconstruction planning, as shown in Figure 1. However, a serious problem with UAV is that their battery is limited and cannot be used for long-term or large-scale survey tasks. Therefore, how to develop a feasible flight path for efficiently and accurately target detection has become a very important issue.

This task mainly includes two aspects, namely path planning [1] and scene understanding [2]. People have done a lot of research on both sides and got some results; however, some problems still exist. For example, most of the previous methods consider this task as two totally-separated problems. Therefore, they may not be well applied to real-world detection tasks in disaster management. In fact, for path planning, the result of scenario understanding is very important feedback. In one single shot, some of the buildings or objects in the picture are not easily discernible and it tends to cause the failure of the scene understanding model. The unclear zone of this area can be used as the key consideration area for the next shooting, based on that, planning the flight path for the UAVs.

Deep learning methods for object detection have been applied in many areas including automatic driving, medical application, urban research, and so on [3,4,5]. These kinds of technology can also be utilized on scenes understanding for path planning. For the flight planning of drones, previous methods mainly considered coverage and total flight time. In our approach, we also need to consider the confidence of the recognition. This is to ensure that the deep learning network [6] can accurately and reliably identify buildings, pedestrians, and other objects in the video data collected by the drone. In the optimization process, the traditional method mainly uses a heuristic algorithm based on the traveling salesman method. This algorithm optimizes the performance of the entire model by minimizing the cost of reaching the current state and the cost of going from the current state to the next state. In our approach, we also chose a similar method to optimize the path. Since we added the elements of confidence, the overall optimization algorithm is more complex.

In this paper, we present an UAV path planning approach for target detection and location tasks using depth models. We want to construct a real-time route planning system by detecting the crucial information. With the information detected, our model can analyze the information density of each area and thus develop an optimal search route. Compared to previous works, our approach achieves more accurate image processing and efficient path planning. The main contributions of our work include:

1. A drone planning algorithm. In this algorithm, based on the traditional heuristic method, we introduce information density. The confidence of object detection can be extracted by a pre-trained detection model and optimized during route planning;
2. By using an object recognition deep learning model. For the video frames captured by drones, our method can simultaneously detect and locate pre-selected objects, such as buildings, pedestrians, and so forth. All the detected information will contribute to the route planning;
3. A drone image dataset. The dataset was collected from real drones. All buildings in each video frame were manually annotated and the exact location mask was marked. It is a first-released dataset for UAV route planning.

The rest of the paper is organized as follows. Section 2 introduces the existing research in related area. Section 3 presents some notations and the problem definition. It gives an overview of the proposed system. In Section 4, we present a scene understanding method and propose the core algorithm of path planning. The experimental evaluation is shown in Section 5. Finally, discussions and conclusions are drawn in Section 6 and Section 7.

## 2. Related Works

### 2.1. Scene Understanding for UAVs

In the early stage of UAVs planning, the performance is limited by the detection accuracy [7,8,9]. Minaeian et al. propose a novel target detection and location scheme, which is based on vision [10] and treats each UAV as a different function of the cooperation team. The authors built a team of UAV and multiple unmanned ground vehicles (UGVs) that track and control the population in the border field. An algorithm, which can detect the custom motion, is used in the crowd detection by a mobile camera installed on a drone. Since a single drone owns low analysis ability but a wide range for detection, UGV has better analysis and higher accuracy. Thus a separate body detector is used and the landmark is moved to locate an unknown independent movement at each point in time. The UAV positioning algorithm proposed in this paper uses a perspective transformation to translate the crowd position in an image into a position in the real world. Moreover, a thumb positioning method determined by UGV is introduced, which makes the prediction for the geographical location in the detection.

The machine learning method seems to be another way to solve the detection problem [11]. Liu et al. propose a framework [12] combining trajectory detection based on support vector machine and tracking tracker to realize direction estimation. It can realize tracking with low computational cost and real-time. In addition, in their system framework, a simple linear iterative clustering super-pixel segmentation algorithm is adopted to ensure the accuracy of scene segmentation. The visual detection of important objects or people is realized by a single-shot multi-box detector algorithm.

How to recognize the target in a spatial dimension is important [13,14,15,16]. Combined with the low-quality attitude-heading reference system (AHRS) on UAVs, Zhang et al. [17] propose a new vision-based positioning method to determine the three-dimensional position of the target. The usual positioning method must rely on many requirements (i.e., geo-referenced terrain database, precise attitude sensor). If the drone system does not meet these requirements, the geographic location of the target cannot be achieved. On the contrary, the location method proposed in this paper only uses computer vision technology to accurately estimate the target height and yaw angle measurement deviation. The purpose of this method is to eliminate requirements in current systems while maintaining high target positioning accuracy. Zhu et al. present a superior estimation method for urban traffic density [18], which can efficiently deal with ultra-high resolution video shots obtained from UAVs. Driving drones during peak hours, they first obtain the traffic video in ultra-high resolution for nearly one hour at five crowded areas in big cities. Then, they randomly sample pixel patches and annotate vehicles to form the data set, which can also be used in other research. In the innovative method of urban traffic estimation, they use a deep neural network to detect vehicles and obtain information, such as location, identification of the vehicle. In addition, they claim that there is other information included in an ultra-high resolution video, making vehicle detection and recognition more accurate than low-resolution content. Fan et al. [19] propose a plant detection method using UAVs. This method consists of three phases. In the first stage, some candidate tobacco plant areas are extracted from the drone image by morphological operation and watershed segmentation. There are tobacco plants or non-tobacco plants in each candidate area. In the second phase, a deep convolutional neural network is established and trained to classify candidate areas as tobacco growing areas or non-tobacco plant areas. In the third stage, post-treatment is carried out to further remove non-tobacco plant areas.

Most of the previous works are not designed for the UAVs nor optimized for detection and localization tasks during flying on the air. It is important to realize the real-time understanding of frames’ contextual information [20,21].

### 2.2. Path Planning of UAVs

When we can well understand the scenes, the next stage is to plan an optimal route. Some works [22,23,24] introduce a solution named collaborative path planning, which utilizes UAV and UGV to track moving targets in urban environments. The most significant advantage of these kinds of algorithms is that it considers the visual occlusion caused by obstacles in the environment. The algorithm models the target state using a dynamically occupied grid, which is refreshed according to the data obtained from the Bayesian filter. Hence, the current behavior and its prediction can be analyzed, based upon which, a single-vehicle path planning method is presented. The method can maximize the sum of detection probabilities and has been applied to various scenarios due to its portability. In this scenario, the auction-based decentralized programming algorithm is designed to plan a limited forward-looking path, maximizing the combined probability of detection and the vehicle. Wen et al. [25] propose a new way to obtain a feasible and safe path. First, based on the intuitionistic fuzzy set (IFS), static threats (STs) are modeled to represent the uncertainty in STs. Based on the fast detection of the random tree (RRT), the reachability set (RS) is supposed to predict the value of threats. Secondly, the main purpose that putting a sub-target selector into the planning scheme is to reduce planning costs and improve the efficiency of searching. Furthermore, a back horizon (RH) is introduced, aiming to deal with online path planning in more complex environments. Therefore, local planners are designed based on the dynamic domain fast detection random tree (DDRRT). And RRT is adopted to achieve the optimization of the path in the planning program. Yin et al. [26] introduce a scheme for multi-objective path planning (mopp), which searches the appropriate path in complex urban scenarios, taking into account the level of safety within the proposed scheme to ensure security of drones. In particular, the security index maps (SIM) are first utilized to detect various obstacles in geographic maps. Thus, the offline search and online search method based on static sim cards are proposed. Offline search is supposed to detect static obstacles, thereby reducing the driving time; however, online search is supposed to deal with other dynamic obstacles. Using the monitoring sensors is another possible way for efficient route planing [27]. Just et al. [28] solve the problem of optimizing the UAV path by considering the sensor nodes to reduce the flight time. This method can greatly improve the efficiency of UAVs planning and maximize the lifetime of sensor nodes.

An evaluation metric is necessary to compare the performance among different methods [29,30,31]. One most utilized way is making statistical comparisons between existing UAV path planning methods to determine the best benchmark function [32,33,34]. Using the approximate optimization technique determined in the first step, namely the multi-verse optimizer (mvo), they formulate the path planning problem of determining the minimum deviation trajectory of the minimum collision of the drone from the mathematical point of view. They also compare with other existing approaches to verify the proposed path planning method. In addition, Yang et al. [35] propose a new approach to individual evaluation and evolution methods. By using this new idea, people can take advantage of high-quality way-points. In the evaluation phase, a new set of evaluation functions is derived from existing targets and constraint functions to evaluate them. Basically, the derivation can only be made if the original function is separable on the way-point. To further improve the performance of the proposed planner, the way-points are encoded in a rotating coordinate system with external constraints. In order to test the ability of new planners in planning barrier-free paths, five scenarios with increasing barriers were constructed. Planners use three existing planners and four alternatives for comparison, which can prove that they can be executed efficiently and effectively.

Most previous works do not consider the prediction confidence when choosing the future path. Therefore, their prediction performance is not so satisfactory during actual tasks.

## 3. Path Planning

**Figure 2:**
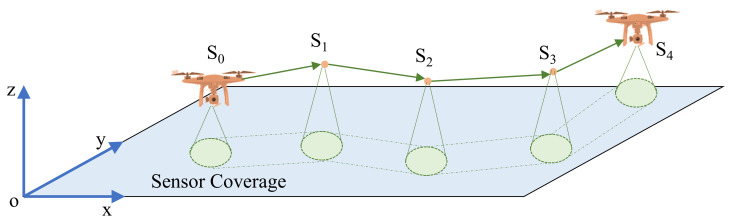
Path Planning for UAVs.

With the development of robotics, the path planning problem of UAVs in a 3D environment is a hot spot. It is one of the basic links in the autonomous navigation of drones and refers to the environment in which obstacles are used according to certain evaluation criteria. The optimal path should be established when facing obstacles. Generally, path planning can be summarized as follows: modeling the environment, executing the path search and building the optimal path. Modeling the environment means abstracting the actual spatial environment information mathematical model. By doing this, the 3D structure information can be processed by computer algorithms. In other words, environmental modeling is path planning, as shown in Figure 2. Consider the 3D environment as an xyz coordinate system. A drone starts from the initial point *o* and reach the first sub-area $S_{0}$. Each drone has its sensor coverage, which represents the capability of the camera. It decides the time needed to fully scan the sub-area. After scanning the $S_{0}$, the drone will fly to the next sub-area $S_{1}$ and continue to do the same operation. The path planning is to find the most efficient path to finish the scanning of the whole area.

Actually, the traditional grid method plays the most basic role among the various modeling methods [36,37]. When modeling the environment, the scenarios are transformed into the traditional grids by which information is beneficial to be preserved in a computer. In addition, the adjacency relationship among the grids is pretty intuitive. It is easy to write a program implementation in practical applications and we can obtain better planning results when combined with the planning algorithm. In the field of robot path planning [38,39], the grid method has been widely regarded by researchers as a typical environment modeling method. With the continuous complexity of environmental information and the continuous improvement of robot autonomy, the grid method is still being used. Compared with the two-dimensional environment, the traditional grid method is used to directly model the environment in the three-dimensional environment. In order to improve the accuracy of modeling, the grid has to be designed into a small size, which leads to a large increase in the number of grids, resulting in the need for an efficient planning algorithm.

This paper mainly considers three kinds of planning indicators, namely the total information density of sub-areas *R*, the coverage time of sub-areas $T_{C}$, and the time of transition $T_{V}$. Suppose a drone or unmanned boat *i* (0≤i≤N) is assigned a sub-areas collection $K_{i}={\left\{k_{ij}\right\}}_{j=1}^{M_{i}}$ ($M_{i}$ is the number of the assigned sub-areas for drone *i*), and the total information density of the *j*-th sub-area is:

(1) $$R_{k_{ij}}=\sum_{o\in Objects}Confidence(o),$$

where the Confidence(o) is the confidence of a detected object by our detection method (will be introduced in Section 4). The sub-area coverage time represents the ideal time required for the drone or unmanned boat to completely cover the sub-area:

(2) $$T_{C,k_{ij}}=\frac{S^{\prime }}{C}\prime$$

where *C* represents the sensor coverage of the drone (this is the area that the drone can scan per unit time), and $S^{\prime }$ represents the subarea area.

The transition time represents the ideal flight required from the initial point $P^{0}$ to the center of the *j*-th sub-area (*j* = 1), or from the center of the j−1 sub-area to the center of the *j*-th sub-area (*j* > 1):

(3) $$T_{V,k_{j}}=\left\{\begin{array}{c} \frac{\left|\right|P^{0}-u_{k_{i(j-1)}}\left|\right|}{V},\;j=1\\ \frac{\left|\right|u_{k_{i(j-1)}}-u_{k_{ij}}\left|\right|}{V},\;j>1.\; \end{array}\right.$$

Among them, *V* represents the speed of movement of the drone and *u* is the center of a sub-area. It depends on the machine and the environment. We consider the speed as *V* under an ideal condition for all the drones.

Therefore, the expected observed benefit of a drone *i* for a sub-area $k_{j}$ is:

(4) $$EP_{k_{ij}}^{i}=\frac{R_{k_{ij}}}{T_{C,k_{ij}}+T_{V,k_{ij}}}.$$

According to the above formulas, the $K_{i}$ sub-areas are iteratively sorted to determine the optimal observation order. It is generally expected that the larger the observed benefit, the higher the information density of the area or the less the ideal observation time, the higher the priority.

After determining the optimal observation order for each sub-area, we get a new sub-areas collection $K_{i}^{\prime }={\left\{k_{ij}^{\prime }\right\}}_{j=1}^{M_{i}}$ with sorting and the expected observed benefits of the drone or unmanned boat i are available:

(5) $$EA_{i}=\sum_{j=1}^{M_{i}}EP_{k_{ij}^{\prime }}^{i}.$$

In addition, define the ideal observation time for each drone:

(6) $$T_{i}=\sum_{j=1}^{M_{i}}\left(T_{C,k_{ij}^{\prime }}+T_{V,k_{ij}^{\prime }}\right).$$

The total allocation indicator for the drone is:

(7) $$EA=\alpha \sum_{i=1}^{N}EA_{i}+\beta \sum_{i,i^{\prime }=1}^{N}|T_{i}-T_{i^{\prime }}|.$$

Among them, the first part represents the total expected observation gain (a higher value means better information efficiency), and the second part is used to balance the task execution cost among the various observation forces such as drones (we want each drone to be distributed tasks evenly). α and β are weight coefficients. The larger the EA, the higher the regional observation efficiency, so the optimal allocation objective is as follows:

(8) max(EA).

After the sub-areas are allocated, each drone or unmanned boat adopts a parallel receding horizon control (RHC) route planning method aiming at maximizing the observation gain, so that the planned route meets the task time constraint. Assuming that the center position of the sub-area to which the drone is assigned is $S_{i}={\left\{s_{j}\right\}}_{j=1}^{M_{i}}$, the route planning procedure based on the parallel RHC is as follows.

First, each route segment is initialized, including the shortest route segment $\phi_{0}$ (time $t_{0}$) from the starting point $P^{0}$ to the center of the sub-area $s_{1}$, and the coverage observation route $\phi_{j}=\left\{s_{j}\right\}$ of each sub-area in $S_{i}$ (time $t_{j}=1$), the shortest transition route $\phi_{j\to j+1}\left(j<M_{i}\right)$ (time $t_{j\to j+1}$) in each sub-area.

Then, if the sum of the time of each of the above-mentioned route segments is less than the task time *T*, then a certain route segment needs to be selected and a new way-point is added. The specific implementation strategy is as follows: adopt the RHC method to pre-plan a new way-point for each route segment $\phi_{j}$, and select from it. The destination point $P_{j^{*}}$ of the largest single observation gain is added to the internal route segment $\phi_{j^{*}}$ of the corresponding sub-area $s_{j^{*}}$, and the time $t_{j^{*}}=t_{j^{*}}+1$ is updated; if $j^{*}<M_{i}$, the slave is updated the point $P_{j^{*}}$ is the shortest transition route $\phi_{j^{*}\to j^{*}+1}$ of the center of the sub-area $s_{j^{*}+1}$, and the flight time. Repeat the above steps to gradually expand each covered route segment until the sum of the time periods is equal to the mission time *T*.

Finally, each route segment is sequentially connected, that is, $\{\phi_{0},\phi_{1},\phi_{1\to 2},\cdots ,$$\phi_{M_{i}-1\to M_{i}},\phi_{M_{i}}\}$, as a planning route.

## 4. Perception Method

### 4.1. Deep Learning Model

**Figure 3:**
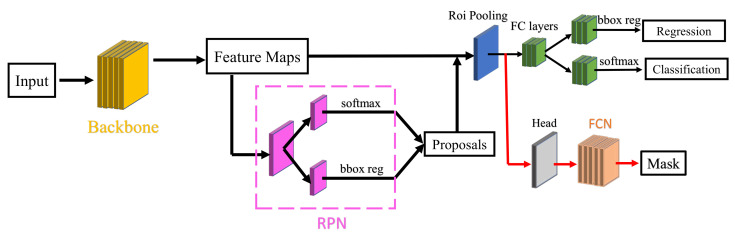
Structure of the adopted Mask-RCNN model.

After obtaining frames of captured video, the system can work on the scene understanding. Compared to other perception methods, which manually design the feature map for the deep model, we directly map the input frames to object categories and localization. Our model is based on Mask-RCNN [40] (the structure is shown in Figure 3).

The ResNet-101 [41] is adopted as the backbone module for the network. We also tried with other network structures (e.g., VGG-16), but the results were no better than ResNet-101. As shown in Figure 3, an input image is first processed by the backbone to generate feature maps. We remove the last pooling layers (pool5) of the original design of ResNet.

The next part is a region proposal network (RPN) [42] for the analysis of the feature maps and proposing candidate building regions (bounding boxes). It estimates the probability of building/non-building on a fixed set of anchors on each position of the feature maps. Meanwhile, the position and size of each anchor are fine-tuned by bounding box regression (bbox reg). After investigating the sizes of the bounding-boxes for buildings, we use three anchor scales (64, 128, and 256) and three anchor ratios (1:2, 1:1, and 2:1) in this study.

The feature maps cropped by the building proposals (256 in our paper) are sent to a region of interest (Roi) pooling layer which will turn all feature maps into a fixed size (7 × 7 in this study). These feature maps are fed into a full-convolutional (FC) layer for further modification. After that, two different FC layers are set to predict the bounding box regression for further fine-tuning and the confidence scores for each building proposal, respectively. Nonmaximum suppression (NMS) [42] is applied to the bounding boxes to decide the final predictions. The intersection-over-union (IoU) thresholds for NMS are 0.7 and 0.1 for training and testing, respectively. Another branch (marked by red) is attached with an FCN [43] model for the semantic segmentation of building masks.

In order to realize the model defined above, a multi-task loss is adopted as the loss function.

(9) $$\begin{array}{c} L=\lambda_{1}L_{\mathrm{category}}+\lambda_{2}L_{\mathrm{regression}}+\lambda_{3}L_{\mathrm{mask}}, \end{array}$$

where $L_{\mathrm{regression}}$ and $L_{\mathrm{mask}}$ are cost functions for two different objectives (localizations and masking), and $\lambda_{1}$, $\lambda_{2}$ and $\lambda_{3}$ can decide their importance for the whole cost value.

$L_{\mathrm{category}}$ represents the loss value to judge the selected data samples as correct category categories, that is, whether or not there is the specific kind of category in the video frames. $L_{\mathrm{category}}$ is indeed a softmax cost function.

There are *n* input images $X=\{x_{1},x_{2},\ldots ,x_{n}\}$ in the deep model, and some different labels for them $x_{i}$, which are called the ground-truth $Y=\{y_{x_{1}},y_{x_{2}},\ldots ,y_{x_{n}}\}$ and localizations $R=\{r_{x_{1}},r_{x_{2}},\ldots ,r_{x_{n}}\}$. The labels can be valued from $y\in \{y_{1}^{*},y_{2}^{*},\ldots ,y_{m}^{*}\}$. *r* is the real localizations value.

(10) $$\begin{array}{cc} L_{\mathrm{category}}\; & =-\frac{1}{n}\left[\sum_{i=1}^{n}\sum_{j=1}^{m}1\left\{y_{x_{i}}=y_{j}^{*}\right\}\log\frac{e^{h_{\mathrm{category}}^{\left(j\right)}\left(x_{i}\right)}}{\sum_{\phi =1}^{m}e^{h_{\mathrm{category}}^{\left(\phi \right)}\left(x_{i}\right)}}\right], \end{array}$$

where $h_{\mathrm{category}}^{\left(j\right)}\left(x_{i}\right)$ belongs to [0,1], representing the confidence that one specific area is a true object, for example, building, car, human, and so forth. *n* represents the number of input samples, *i* is the sample id of one input sample, *m* represents the total category number, *j* is the specific category id, $h_{\mathrm{category}}\left(x_{i}\right)$ is the Softmax result, and $h_{\mathrm{regression}}\left(x_{i}\right)$ is the Smooth $L_{1}$ result.

$L_{\mathrm{regression}}$ is utilized to find the object localization of one object. According to the smooth $L_{1}$ loss in of Faster-RCNN [42], $L_{\mathrm{regression}}$ can be written as:

(11) $$\begin{array}{c} L_{\mathrm{regression}}=\frac{1}{n}\sum_{i=1}^{n}{\mathrm{smooth}}_{L_{1}}\left(h_{\mathrm{regression}}^{\left(r\right)}\left(x_{i}\right)-r_{x_{i}}\right). \end{array}$$

$L_{\mathrm{mask}}$ is calculated in the same way defined by Mask-RCNN [40], which aims at semantic prediction for each foreground object.

### 4.2. UAV Image Dataset

We created a new drone image dataset ourselves to train our deep neural network. The training set for neural networks comes from the video that is actually captured on the drone. We extract key frames for it, and then perform image calibration and area marking on each image. The marking tool we chose is LabelMe. LabelMe is an annotation markup software made by Python and drawn with GUI by Qt. It can greatly improve the efficiency of image marking.

We have marked a total of 1000 drone images, with dozens of buildings to be marked on each image. The entire marking process took three months/person and marked more than 30,000 different types of buildings with different appearances. Ten percent of the random split data is used to form the test set and the rest are used for training the model. For all the experiments, we trained our model with 10,000 iterations using a batch-size of 4.

**Figure 4:**
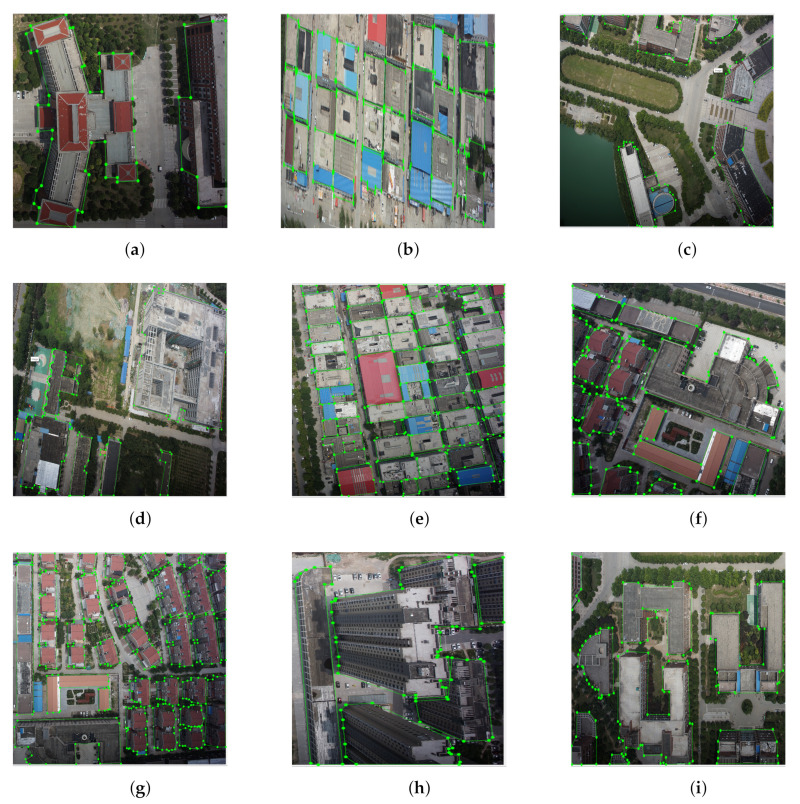
Labeled UAV dataset.

The marked image is shown in Figure 4. Figure 4a–i are the samples from our UVA dataset. Each building has clear boundaries and tag values. The annotators are required to use poly-point to annotate each foreground building. The LabelMe software will automatically generate the bounding boxes and masks under the structure of the COCO dataset. In the experimental part, we used this database for training and testing, and some of the results are shown in the next section. Our best performance is achieved by using Mask-RCNN with ResNet-101 as the backbone and the total model size is 358.46 M.

## 5. Performance Evaluation

### 5.1. Recognition of Building Objects

The training process is performed with some state-of-the-art deep learning frameworks by detectron2 [44] and a modernized NVIDIA GTX 1080 GPU. The learning procedure takes about 2 h due to the super-large data. We tried our network with a different backbone and the best mAP of 0.921 on the split test set was achieved by ResNet-101. Thus, the results of the continued experiment are shown with this setting. As shown in Table 1, we also compare the adopted method to other object detection methods. We want to compare the performance of different detection models for our dataset. We can observe that the proposed method achieves the best mAP performance. Therefore, the proposed detection method can better serve the Confidence(o) defined in Equation (1). In addition, we present some instances for the results of confidence value and visualization in the Subsections.

### 5.2. Confidence Value

**Figure 5:**
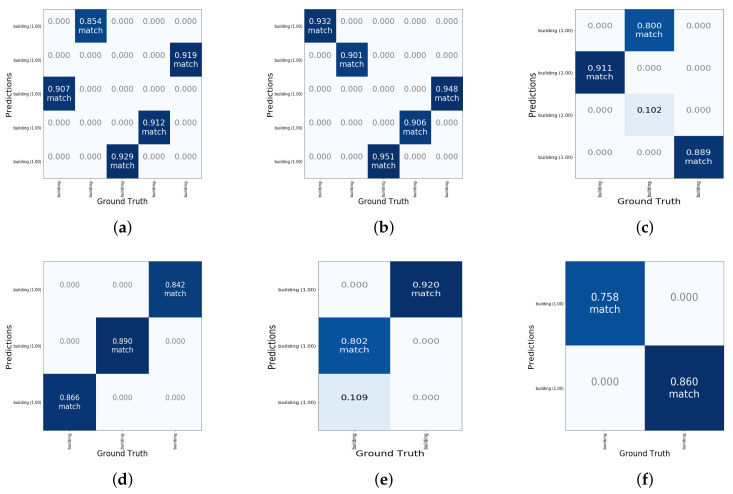
Grid of ground truth objects and their predictions.

As mentioned above, the object is divided into different grids and is detected. We analyzed the data obtained from the input image and made the prediction of whether it is a building. In Figure 5, Figure 5a–f are the samples of performance evaluation of out method, we show six results for the grid of ground truth objects (buildings) and their predictions. Some examples have 25 grids while some examples have nine grids, eight grids, or other numbers. The number of grids depends on the number of buildings in the background. The more buildings for ground truth, the more grids should be generated. For example, if the model predicts *n* building objects and the ground truth is *m*, we will show the result with a n×m matrix which divides the image into grids. According to the coordinate predicted by the model or ground truth, we can calculate the center of each building and assign them to each grid. Generally, the grid colored with blue represents that there is a building in the real world, and the other grid means there is not a building in the real world. As for the value of each grid, it is the confidence value for the prediction. In our method, we estimate that there is a building if the confidence value exceeds 0.6. The possibility that there is a building is high when the confidence value is higher. The word *match* drawn on the grids means the prediction is correct corresponding to the ground truth.

For example, in Figure 5a, the confidence value of the first grid in line 1 is 0, which is lower than 0.6. Therefore, we make the prediction that there is no building in this grid. The confidence value of the second grid in line 1 is 0.854 which is larger than 0.6. Then we make the prediction that there is a building in this grid, which is proved to be true according to the ground truth results. In general, a high confidence value in the grid means that there is possibly a building in the real world. By using this kind of grid evaluation, we can directly relate the detection accuracy to the route planning, which is also based on the grid division.

### 5.3. Visualization

**Figure 6:**
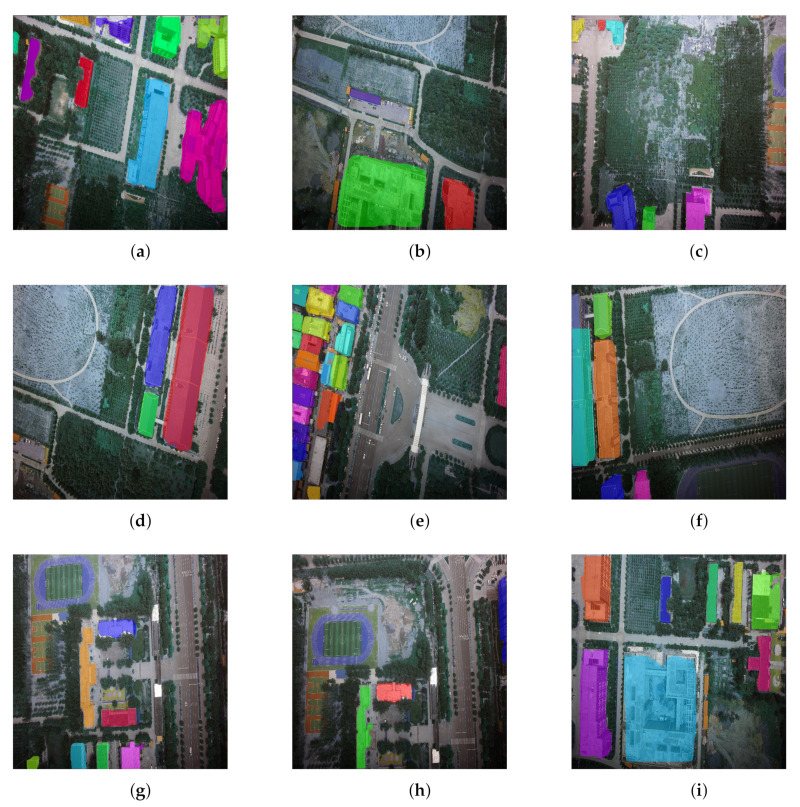
Visualization of the building predictions.

Figure 6a–i illustrate the visualization outcomes of the building predictions. The background is the irrelevant things (e.g., tree, land) for the real scenario in each instance, and the identified buildings are labeled with different colors. We show the visualization outcomes for nine scenarios, including the commercial circle, the playground and the road area. The buildings are dense in some scenarios, and others are not, which is related to the number of grids generated in the method. Among the nine visualization outcomes, it can be seen that the size and shape of the building can be drawn with the colored areas. In general, our method has recognized all the buildings and has high performance; however, there exist some points to be improved. For example, there are ten buildings that are recognized in Figure 6a and labeled with blue, purple, green, and so on. However, some corners and boundaries are not completed in the visualization outcome when we check the results in detail. Moreover, some results show errors in terms of recognizing the wrong object. In Figure 6g, for example, it can be seen that the playground has been colored purple. However, the playground is not a building. And in Figure 6i, the ring building, colored blue, is not recognized accurately enough. The reason is possibly that the center area in the ring building is not a part of the building.

According to the results of comparison between ground truth and predictions in grids as well as the visualization for building predictions, our method has achieved high performance.

## 6. Discussion

### 6.1. Analyse of Detection

As discussed in Section 5, our detection model owns high performance. However, there are still some mistakes in the building prediction. We will analyze them in terms of the following aspects:

**Size of Anchor Generation** We introduced the model structure in Section 4.1. The RPN layer will physically generate the anchor for detecting building objects. We default the initial anchor size as 64, 128, and 256. Generally, this setting covers most of the building size on the image. However, there is also the situation that building as densely distributed (e.g., Figure 4). In this scene, some buildings can not be detected or even multiply buildings detected as one entity.

**Confusing Background** There are some background things similar to buildings. They will cause the prediction error during the detection. As shown in Figure 6g,h, the playground is detected as a building and it is meaningless for a drone to search that area. We find this error can be decreased by improving the confidence threshold set. Thus, we set the confidence value as 0.6 which gives the best detection performance.

**Scanning Distance** The distance when drone capturing the images is really important for the detection task. When giving the high perspectives, even a large building turns small, which may make the information density bigger. In our dataset, we set a fixed camera height to avoid this problem.

### 6.2. Computing Cost and Possible Application

For the inference of detection, the computing time for each image is 0.118 s on average (shown in Table 1). This time can be shorter by using better GPU or decreasing the number of proposals. However, the current running speed is enough for the real-time application. For the computation of path planning, it depends on how many grids are divided and how careful the searching is, which is a trade-off for UAVs problems.

Our approach is designed for real-world tasks and can be applied in many scenarios. Considering the situation that there is fire in the community, a highly accurate and efficient UAVs search is necessary. It can firstly detect all the foreground objects and plan the best way for searching. During the search, the route can be further modified due to the changes of the terrain or the people. We will test our method in a real-world experiment in the future.

### 6.3. Limitation

An important factor for this method is the signal communication between the drone and local server. Our model needs the computation power of GPU to realize real-time detection. It is not practical to put a GPU on a drone. Thus, the images captured by the drone need to be transferred to the local server. After computing, the detection can be fedback to the drone. A good communication environment is important. Thus, it may not work well in some extreme weather (e.g., thunderstorm). Another thing that needs to be discussed is the image quality captured by a drone. Our method is highly dependent on the results of building detection. A good camera is necessary. Even on the same scene, different camera resolutions will show different imaging results. Some small buildings may be difficult to recognize when the resolution is poorer. However, higher resolution brings additional computational consumption.

*The flight time of the drone* and *coverage of the area to be detected* are dependent on the machine and the environment. We consider the speed as *V* under an ideal condition for all the drones. Confidence(o) of object detection depends on the model performance and the quality of captured images. Thus, our method is based on an ideal situation and those three points cannot be guaranteed. However, it could work for most real-world situations. For the optimization, the object detection uses a pre-trained model. Thus, path planning is the only thing that needs to be computed. There may be a possible way to optimize them at the same time; however, this needs to be further explored.

## 7. Conclusions

In this paper, we designed a new fly path task for UAVs, considering the actual perception needs. A convolutional neural networks (CNNs) model is proposed to detect and localize the objects, such as buildings. Taking the object confidence as a computing factor, we proposed an optimization method to find the optimal flying path to realize effective and efficient detection with a minimum time cost. To evaluate our detection method, we also prepare a drone image dataset collected from real drones. The results demonstrate that our detection model can realize high accuracy object recognition. We think our method has the potential to address the actual scene understanding and path planning problems. We will further improve the current algorithm and explore the application of the proposed method for real-world UAVs tasks in the future work.

## Figures and Tables

**Table 1:** The performance of different object detection methods in our dataset.

Method	mAP	Speed
YOLO-V3 [45]	0.791	0.082 s
DETR [46]	0.865	0.455 s
RetinaNet [47]	0.823	0.217 s
EfficientDet [48]	0.917	0.112 s
Ours	0.921	0.188 s

## Data Availability

The data presented in this study are available on request from the corresponding authors.
